# Randomised crossover trial of telemonitoring in chronic respiratory patients (TeleCRAFT trial)

**DOI:** 10.1136/thoraxjnl-2015-207045

**Published:** 2016-03-09

**Authors:** M Chatwin, G Hawkins, L Panicchia, A Woods, A Hanak, R Lucas, E Baker, E Ramhamdany, B Mann, J Riley, M R Cowie, A K Simonds

**Affiliations:** 1 NIHR Respiratory and Cardiology Biomedical Research Units, Royal Brompton & Harefield NHS Foundation Trust, London, UK; 2 Department of Basic Medical Sciences, St Georges Hospital, London, UK; 3 Department of Respiratory Medicine, West Middlesex University Hospital, Isleworth, Middlesex, UK

**Keywords:** COPD Exacerbations, Respiratory Infection

## Abstract

**Objective:**

To assess the impact of home telemonitoring on health service use and quality of life in patients with severe chronic lung disease.

**Design:**

Randomised crossover trial with 6 months of standard best practice clinical care (control group) and 6 months with the addition of telemonitoring.

**Participants:**

68 patients with chronic lung disease (38 with COPD; 30 with chronic respiratory failure due to other causes), who had a hospital admission for an exacerbation within 6 months of randomisation and either used long-term oxygen therapy or had an arterial oxygen saturation (SpO_2_) of <90% on air during the previous admission. Individuals received telemonitoring (second-generation system) via broadband link to a hospital-based care team.

**Outcome measures:**

Primary outcome measure was time to first hospital admission for an acute exacerbation. Secondary outcome measures were hospital admissions, general practitioner (GP) consultations and home visits by nurses, quality of life measured by EuroQol-5D and hospital anxiety and depression (HAD) scale, and self-efficacy score (Stanford).

**Results:**

Median (IQR) number of days to first admission showed no difference between the two groups—77 (114) telemonitoring, 77.5 (61) control (p=0.189). Hospital admission rate at 6 months increased (0.63 telemonitoring vs 0.32 control p=0.026). Home visits increased during telemonitoring; GP consultations were unchanged. Self-efficacy fell, while HAD depression score improved marginally during telemonitoring.

**Conclusions:**

Telemonitoring added to standard care did not alter time to next acute hospital admission, increased hospital admissions and home visits overall, and did not improve quality of life in chronic respiratory patients.

**Trial registration number:**

NCT02180919 (ClinicalTrials.gov).

**Key messages**

**What is the key question?**

- Does telemonitoring reduce healthcare demands and improve health-related quality of life in patients with severe chronic respiratory disease?

**What is the bottom line?**

- Telemonitoring did not delay time to next acute hospital admission for an exacerbation, increased health service usage and did not improve quality of life.

**Why read on?**

- To find out why telemonitoring may increase healthcare activity.

## Background

Telemonitoring has been advocated in a variety of chronic conditions including chronic heart failure, diabetes, and COPD, with the goal of reducing hospital admissions, improving self-care and enhancing quality of life. In patients with chronic heart failure, a systematic review and meta-analysis^1^ suggested that remote home monitoring reduced admissions to hospital and all-cause mortality while improving quality of life, but subsequent larger randomised controlled trials in the USA^2^ and Germany^3^ showed no advantage to the addition of telemonitoring to usual care on a range of primary endpoints including mortality and all-cause admissions.

COPD affects around three million individuals in the UK and is the second commonest cause of hospital admission. A third of patients are readmitted within three months of discharge following an acute exacerbation.^4^ A Italian randomised trial^5^ of tele-assistance in chronic respiratory patients requiring long-term oxygen therapy (LTOT) or home mechanical ventilation showed 36% fewer hospitalisations or general practitioner (GP) calls, and reduction in healthcare costs in the telemonitoring group, with the effect more pronounced in patients with COPD. In Bilbao, Spain, telemonitoring in COPD and heart failure resulted in a greater proportion of patients without an admission over 12 months.^6^ A Cochrane review and meta-analysis of telemonitoring in COPD in 2012^7^ found a decrease in hospitalisations and visits to the emergency room but no impact on quality of life. This was followed by the Whole Systems Demonstrator (WSD) trial in the UK, which compared usual care versus addition of telemonitoring in patient groups with COPD, heart failure and diabetes. Initial headline reports^8^ showed a reduction in mortality, emergency admissions, bed days and tariff cost, but subsequent analysis found no change in quality of life for patients, and no quality-adjusted life-year gain from telemonitoring compared with standard care alone.^9^

More recently, a randomised trial of telemonitoring versus usual care using a broadband system in COPD patients in Scotland showed no change in time to admission for next exacerbation, frequency of admissions or quality of life with telemonitoring.^10^

Despite these negative outcomes, telecare is being advocated to assist a shift from acute hospital management to greater care in the community and has already attracted considerable health service investment.^11^ We reasoned that the addition of telemonitoring in a group of severe respiratory patients might reduce healthcare activity by improving the patients’ ability to identify exacerbations and carry out self-care. So in order to clarify the role of telehealth in a severe chronic respiratory cohort, we carried out a trial of telemonitoring to determine effects on hospitalisation, healthcare utilisation and quality of life.

## Methods

The TeleCRAFT trial (ClinicalTrials.gov identifier NCT02180919) was a randomised crossover study with 6 months telemonitoring and 6 months control period. Patients were recruited from outpatients and inpatients at Royal Brompton & Harefield NHS Foundation Trust, London SW3, West Middlesex University Hospital, Hounslow, Middlesex, TW7, and St George's University Hospital, London, SW17, over the period July 2009 to July 2011. Management was carried out by a combination of allied health professionals, predominantly clinical nurses in the hospital who were aware of allocation to telemonitoring or control group and who liaised with primary care teams. Analysis was carried out blind to trial limb participation. Telemonitoring occurred during the hours 09:00–17:00 Monday through Friday, similar to the protocol of Vitacca *et al*.^5^ Outside these hours, patients continued to use their personalised management plan and could call the on-call senior respiratory hospital doctor or primary care team for further advice.

### Patients

We recruited patients aged 18 years and above with COPD or chronic respiratory failure due to another chronic respiratory disorder (eg, bronchiectasis, chest wall disease, neuromuscular disorder), who had been admitted with an infective exacerbation of their chronic lung disease within the previous six months and who fulfilled criteria for LTOT^12^ or who had an arterial oxygen saturation (SpO_2_) level of ≤90% on air during the previous admission. The rationale for this was to enable home SpO_2_ monitoring to be a realistic marker of acute deterioration in clinical status. The diagnosis of COPD or respiratory failure due to another cause was confirmed by a specialist respiratory physician.

**Figure 1:**
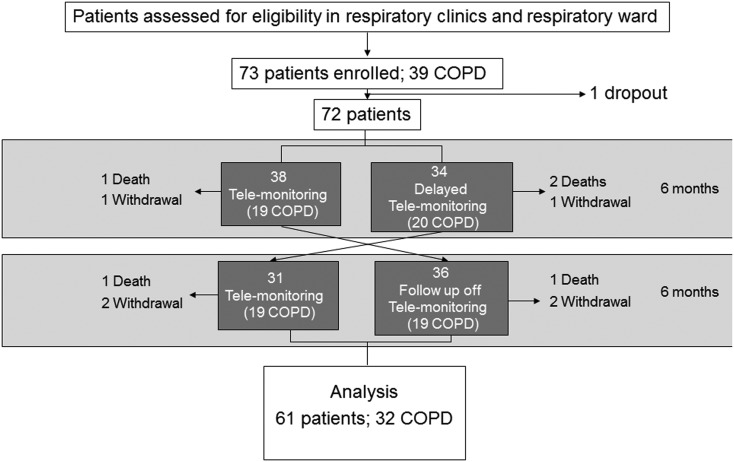
Consort diagram; analysis was on an intention-to-treat basis.

Exclusion criteria were cognitive impairment sufficient to impair understanding of the trial or interfere with use of telemonitoring, and age <18 years. Patients meeting eligibility criteria who consented to participation were included in the trial (see consort diagram in figure 1). An exacerbation was defined as a sustained worsening in respiratory condition, beyond day-to-day variation, necessitating a change in therapy.

Patients were randomised to telemonitoring or delayed telemonitoring (control group), stratified for COPD or non-COPD diagnosis in blocks of five with results generated and made available from our statistics unit. Prior to randomisation, treatment was optimised including non-invasive ventilation (NIV) settings and O_2_ flow rate where applicable, to ensure stability as far as possible.

Patients with chronic heart failure were recruited in a parallel telemonitoring trial; this cohort was not part of this respiratory crossover trial, and results will be reported elsewhere.

The primary outcome measure was time to first hospital admission for an acute exacerbation. Exacerbations were characterised as defined either as a self-reported exacerbation; an episode of taking antibiotics and/or steroids in the control limb, or in the telemonitoring limb a decrease in SpO_2_ with or without a change in sputum production or colour or increase breathlessness that prompted antibiotic and/or steroid therapy. Hospital admissions for respiratory exacerbations were confirmed by review of the hospital discharge summary or the patient's notes.

*Secondary outcome measures* were hospital admissions, GP consultations, home visits and hospitalisations for non-respiratory causes, expressed as rates per 6 months. We also assessed aspects of health-related quality of life by the hospital anxiety and depression score (HADS),^13^ EuroQol-5D (EQ-5D)^14^ and self-efficacy using the Stanford self-efficacy scale.^15^ Hospital and health visits were tallied at the end of each month from patient diary cards (control limb) and information logged into the telemonitoring system (telemonitoring limb). Any query on the nature of healthcare visit in both trial limbs was clarified by follow-up phone call. HADS, EuroQol 5D and self-efficacy score were measured at 0, 3 and 6 months in each trial limb.

*Telemonitoring* was carried out in the patient's home using the Philips Motiva system (Phillips Healthcare, Guilford, Surrey, UK) that comprises heart rate monitoring, finger pulse oximeter, weight scales and blood pressure monitor. The system requests daily responses to a questionnaire on breathlessness, wheeze, sputum production, sleep quality and therapy alterations, for example, a course of prednisolone started, LTOT flow rate increased. The symptom questionnaire responses, heart rate and SpO_2_ were recorded daily, and blood pressure and weight were measured once a week. These inputs were linked to the patient's television screen as an additional television channel that was accessed by hand-held remote. Each patient received education by the Philips Motiva team in using the monitoring devices until he or she and/or family/carer felt fully confident using it. Patients/careers completed a satisfaction questionnaire on ease of use.

Data from the monitors were delivered to the healthcare team members’ personal computer using a dedicated broadband line that was installed in each patient's home and routed via a secure Philips Server. Results were reviewed daily by a healthcare team member (Monday–Friday) by 14:00. These results from each patient daily questionnaire, heart rate and SpO_2_ reading were used to create an individualised traffic light warning system such that if all parameters were satisfactory this appeared as a green light; or, for example, if a drop in SpO_2_ and increase in wheeze, or increased breathlessness is seen, the telemonitoring system generated a red light for that patient, which required action by monitoring staff. The system is interactive, and therefore, the healthcare team member can advise the patient through their screen or by telephone (eg, start antibiotic, increase bronchodilator use), or provide educational material, for example, on inhaler use or exercise; and patients can respond. Any SpO_2_ or heart rate reading outside the patient's preset set limits was compared with the patient's previous data and linked to symptom changes. Therefore, actions depended on a combination of symptoms, telemonitoring results and clinical judgement. If the nurse was not sure what action to take, the advice of a hospital consultant or GP was sought. If patients failed to respond to measures suggested by initial telephone advice, or their condition was deteriorating rapidly, they were advised to contact their GP (and management discussed with GP). If it was deemed more appropriate by the telephone consult, a home visit by the patient's community/primary care nursing team or hospital visit was arranged. Via the telemonitoring system, patients were questioned weekly on whether they had consulted their GP, had a home visit, attended the Accident and Emergency Department or been admitted to hospital. For overnight and weekend health problems, each patient continued to use a personal management plan as described below. Where weekend exacerbations were reported, a phone call was carried out the following Monday.

### Standard care

Patients were managed in accordance with conventional guidelines.^12^ ^16^ Each patient was provided with a rescue pack (antibiotics and prednisolone) where indicated. Patients with COPD had already completed a course of pulmonary rehabilitation and, where indicated, had undergone a smoking cessation programme. Any patient on NIV underwent a sleep study to optimise ventilator settings. Oxygen was provided in accordance with current guidelines.^12^ ^16^ Each patient had a personalised home care plan for escalating therapy with instructions regarding antibiotic therapy, corticosteroid treatment and adjustment of inhaled medication. Patients were provided with a contact number of their medical team and also had ready access to respiratory care nurses that were part of the trial, and their GP.

### Sample size calculation

To assess time to hospital admission for an acute exacerbation (first readmission), we used the results of Vitacca *et al*^5^ and previous experience. To detect a difference of 25% in median time to admission (median 120 days vs 150 days), using within-patient SD of 60 days, significance level 0.05 and power 0.8, a sample size of 63 is required. To allow 10% dropout rate, 69 patients are required.

### Analysis

Paired t tests were used to compare hospital admissions and secondary outcome measures during control and telemonitoring limb. Further, McNemar's tests were conducted stratifying by COPD and respiratory failure groups to investigate potential group differences. Unpaired t tests were used to compare periods (‘control first, telemonitoring second’ and ‘telemonitoring first, control second’ limbs). Results that were not normally distributed are presented as median and IQR.

## Results

### Trial recruitment

Seventy-three respiratory patients were recruited, the majority at Royal Brompton & Harefield NHS Foundation Trust, of which 39 had COPD and 34 chronic respiratory failure. Recruitment began in July 2009, and the trial was completed in July 2013. Seventy-two patients were randomised, 38 (19 COPD) to telemonitoring first and 34 (20 COPD) to control group first. As per consort diagram (figure 1), 67 patients completed the first limb and at 12 months, analysis was completed in 61 patients (32 COPD). Baseline demographics of patient groups are shown in table 1. Individual baseline demographics for each patient can be found in online supplementary data. Comorbidities are shown in table 2.

**Table 1:** Baseline demographics

	COPD	Non-COPD	All
	Mean (SD)	Mean (SD)	Mean (SD)
Age (years)	65.3 (7.6)	58 (14.4)	61.8 (11.9)
FEV1 (L)	0.9 (0.5)	0.7 (0.5)	0.9 (0.5)
FVC (L)	2.1 (0.9)	1.1 (0.6)	1.7 (1)
PaO_2_ (kPa)	8.4 (1.2)	8.2 (1.4)	8.3 (1.3)
PaCO_2_ (kPa)	6.4 (1.3)	6.9 (1.3)	6.7 (1.3)
SpO_2_ (%)	92 (3)	89 (6)	90.4 (4.9)
MRC dyspnoea scale	4 (1)	4 (1)	4 (1)
Gold (stage)	3 (1)	NA (NA)	NA (NA)
Height (cm)	167 (8)	160 (12)	164 (11)
Weight (kg)	85 (26)	83.1 (38.2)	84.4 (31.9)
BMI (kg/m^2^)	31 (9)	33.9 (14.3)	32 (11.5)
HADS anxiety score	8 (4)	10 (4)	9 (4)
HADS depression score	7 (4)	8 (4)	8 (1)
EQ-5D scale score	57 (16)	57 (21)	57 (18)

	N (%)	N (%)	N (%)

Males	20 (63)	9 (32)	29 (48)
NIV	27 (84)	25 (89)	52 (87)
LTOT	19 (59)	19 (68)	38 (63)
Lives alone	9 (28)	10 (36)	19 (32)

BMI, body mass index; HADS, hospital anxiety and depression score; LTOT, long-term oxygen therapy; MRC, Medical Research Council; NIV, non-invasive ventilation.

**Table 2:** Comorbidities

Comorbidity	Amount (n)
Heart failure	9
Diabetes	14
Chronic renal failure	0
Hypertension	26
CVA/TIA	1
Myocardial infarction	1
Angina	1
Pulmonary hypertension	4

CVA, cerebral vascular accident; TIA, transient ischaemic attack.

### Hospital admissions for exacerbations

**Figure 2:**
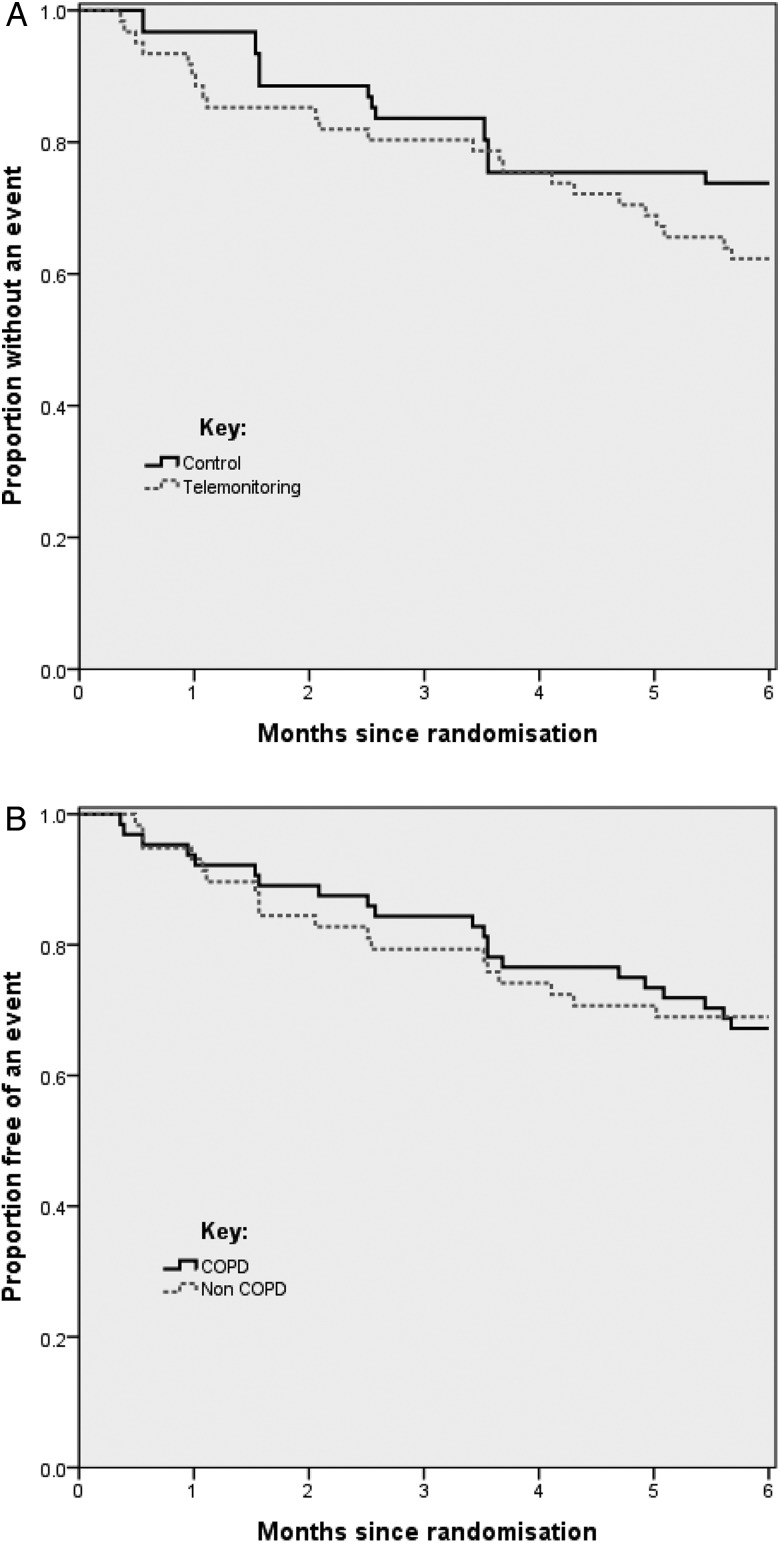
(A) Time to first exacerbation requiring hospital admission telemonitoring versus control for the overall results. (B) Time to first exacerbation requiring hospitalisation in COPD and non-COPD groups.

Time to first acute respiratory exacerbation requiring hospitalisation did not differ between telemonitoring and control limb overall. Of the subjects who experienced an event in both limbs, the median (IQR) days to hospitalisation was 77.5 (61) versus 77 (114) in control and telemonitoring groups, respectively (McNemar's test p=0.189). Figure 2A shows a Kaplan–Meier plot of the time free of hospitalisation in the control and telemonitoring groups. Figure 2B, which shows a Kaplan–Meier plot of the time free of hospitalisation in the patients with COPD and non-COPD patients, indicates no difference between these subgroups.

Respiratory admissions for acute exacerbations at 6 months increased in the group receiving telemonitoring—frequency 0.32 control versus 0.63 telemonitoring (mean difference 0.32, p=0.026), although this number is small (table 3). There was no evidence of a difference between patients with COPD and non-COPD patients for respiratory admissions (p=0.955).

**Table 3:** Six-month healthcare activity

		Control	Telemonitoring	Difference	
	N	Mean	Mean	Mean (SE)	p Value
Respiratory hospital admissions	57	0.32	0.63	0.32 (0.14)	0.026
Home visits	52	0.75	4.00	3.25 (0.81)	<0.001
Hospital visits	49	3.23	3.79	0.56 (0.65)	0.394
GP visits	49	5.17	5.75	0.58 (1.03)	0.573

GP, general practitioner; N, number in analysis.

Home visits over 6 months increased (frequency 0.75 control vs 4.00 telemonitoring, p<0.01) during the telemonitoring period, but there was no change in frequency of GP visits and no difference in hospital visits or non-respiratory cause hospital admissions between trial limbs (p=0.933). The results of unpaired t tests provide support for the assumption of no period or order effect, with no evidence to suggest a period effect for primary or secondary outcome variables.

EQ-5D scores (in total and for individual questions) and EQ-5D scale did not differ between groups, HAD anxiety score was also similar but HAD depression score showed overall improvement in patients receiving telemonitoring compared with the control group (table 4). However, these differences are small and occur in patients in whom depression score is within the normal range and do not exceed the minimally clinical important difference (MCID) in other clinical circumstances.^17^ In a subgroup with a HADS depression score of >11, there was a greater fall during telemonitoring but the number in this group was small and so of doubtful clinical value.

**Table 4:** Health-related quality of life and self-efficacy scores

		Control	Telemonitoring	Difference	
	N	Mean	Mean	Mean (SE)	p-Value
HAD anxiety score	47	7.98	8.25	−0.28 (0.3)	0.364
HAD depression score	47	6.9	6.37	0.53 (0.26)	0.046
Stanford score	46	5.82	5.18	0.64 (0.19)	0.001
EQ-5D scale score	53	59.6	57.85	1.75 (1.57)	0.270

HAD, hospital anxiety and depression score; N, number in analysis.

### Stanford self-efficacy score

The self-efficacy score declined when telemonitoring was used in comparison to the control group for the total patient cohort (table 4). However, there was no evidence of a difference in the effects in the COPD and non-COPD groups (p=0.290). The MCID of Stanford score is not well established in this patient group, so clinical relevance may be minor. However, at best, there is no evidence that the patients’ self-efficacy improved during telemonitoring.

There was a relatively low mortality rate. Five patients died (see figure 1). All deaths occurred in an acute respiratory ward during an exacerbation. Each of these patients had an agreed ceiling of care of NIV, apart from one patient with a Do Not Attempt Resuscitation order who had a cardiorespiratory arrest and died in the Emergency Room. There was no difference between the telemonitoring and standard care groups.

All patients successfully used the telemonitoring system, and in no case did we detect that telemonitoring delayed admission, or caused harm. Via the questionnaire, all but two patients reported using the equipment after demonstration ‘was not a problem’; the remaining two found the instructions ‘a little hard to follow’.

Table 5 shows the number of telephone consults and the alerts generated per month. The majority of alerts were related to SpO_2_ levels.

**Table 5:** Number of telephone consults and patient-related alerts generated

Consults and alerts	Number per month
Total telephone consults	29
Total telephone consults for low SpO_2_	18
Total alerts due to SpO_2_	187
Total ‘red’ alerts due to SpO_2_	31
Total out-of-range tasks	337
Total ‘red’ alerts	460

## Discussion

This randomised crossover trial showed telemonitoring did not lengthen time to next exacerbation requiring acute hospitalisation; indeed, admission rate for acute exacerbations and home visits increased. Quality of life measured by EQ-5D generic scores did not improve during telemonitoring and self-efficacy fell during the trial but particularly in those using telemonitoring first. The only improvement was a lower HADS depression score during telemonitoring, although the significance of this is questionable as it represents a change of only 0.53 units in patients who, on average, were not clinically depressed and is less than the minimal clinically important difference.^17^ HAD anxiety score did not change.

### Interpretation of findings in the context of previously published work

The present study showed an increase in healthcare activity generated by telemonitoring, which was not expected. Although time to first readmission for acute exacerbation did not change, actual admissions doubled from 18 to 36 and there was a substantial increase in home visits. We acknowledge that the criteria for hospital admission are dependent on local hospital policy as not all patients were directly admitted to our centre. However, like Pinnock and coworkers,^10^ we cross-referenced all admissions with discharge summaries to ensure that admission was due to an acute exacerbation. Home visits were carried out if the patient was not responding to telephone advice. The explanation for this increase in healthcare activity including home visits and the absence of reduction in hospital admissions seen in other telemonitoring trials may be due to the reduction in self-efficacy. This may cause patients to rely more heavily on the telemonitoring team who instigated more reviews both using home visits and hospital visits. Indeed, the potential for telemonitoring to increase reliance on healthcare professionals has also been reported in COPD and asthma by others.^18^ ^19^ This is the opposite of the intended effect of improving patients’ ability to self-care, thereby reducing admissions and healthcare activity, and improving quality of life. The main triggers for a home visit were fall in SpO_2_, accompanied by symptoms. Variation in SpO_2_ can be helpful in predicting exacerbations and may be valuable in patients who fail to recognise the development of an exacerbation, and therefore, seek treatment late.^20^ Predictive composite scores of heart rate variation and SpO_2_ have been developed in patients with COPD with mild desaturation at rest.^21^ By selecting more severe patients using LTOT or having an SpO_2_ <90% on air during index admission in the TeleCRAFT trial, this baseline hypoxaemia (SpO_2_ mean (SD) 92 (3)% in patients with COPD and 89 (6)% in non-COPD patients) meant that more marked variation in SpO_2_ occurred as SpO_2_ was on the steep descent of the oxygen dissociation curve. Our expectation was that day-to-day variation in this more hypoxemic group will be greater, making it more likely that intervention would be prompted by the healthcare team observing daily data. A better understanding of day-to-day variation of SpO_2_ in this severely compromised respiratory population would be helpful and might decrease home visits and hospitalisations. However, patterns of exacerbation tend to show more variation between individuals rather than within individuals, making algorithms in practice only weakly predictive.^22^ Teams may gain more understanding of diurnal variation in variables as their experience with telemonitoring in individual users increases. This finding also exemplifies the fact that more data can be helpful, but can also add to clinical uncertainty and therefore precipitate more activity. Increased healthcare activity and home visits in turn may act as a deterrent to the patient taking responsibility for his/her disease and may lead to co-dependency between patient and healthcare team.

### Limitations of study

Our patients are of a similar age to that of Vitacca and coworkers,^5^ and we included a similar proportion using long-term NIV. This extent of NIV use may not occur in other units. We ensured all patients were optimally treated with NIV prior to entering the study and no patient started LTOT or NIV during the study. Although the age is slightly younger than that in the trial of Pinnock and coworkers,^10^ COPD recruits had a similar mean GOLD stage. The majority of patients were recruited within the first two months after a hospital exacerbation. Our patient group was characterised by being hypoxaemic during an exacerbation or on LTOT. We selected this group of patients that were hypoxic on admission or on LTOT with the hypothesis that oximetry would be the most helpful and discriminating observation. Most importantly, this group represents our clinical case load and we wanted to evaluate the role of telemonitoring in this group. While we still feel that our results are applicable elsewhere, the findings cannot be generalised to those with milder chronic respiratory disease who are normoxaemic.

The sample size was calculated at 63, and while 72 patients were entered, 61 patients completed the study. This reduces the power of the trial, but it seems unlikely that additional patients would have altered the outcome.

A perceived limitation of the study is that team members managing the patients knew whether they were in telemonitoring or control limb as they had telemonitoring data available to them, so were not blinded to the intervention. However, like Vitacca and coworkers,^5^ we monitored our patients for 40 h per week. Our patients also had contact telephone numbers of community team members for contact at weekends and evenings. Unlike the Vitacca *et al*^5^ trial, our telemonitoring team knew the patients and were familiar with their respiratory status. We felt that this would help prevent unnecessary home visits or over-identification of exacerbations. It maybe also be seen as a limitation not having 24/7 care, but one of the aims of telemonitoring is to promote independence and self-management rather than reliance on healthcare professionals. This outcome was not supported by the results of this study. The fact that the control group had access to phone support may not be standard in other centres and could mitigate any additional effects of telemonitoring.

A further criticism of the present study is that we did not include a washout period. However, the aim of the present study was for the patient to act as their own control to see whether education and management during telemonitoring improved self-efficacy. Therefore, we do not believe that there is a sensible length of washout period as patients may or may not gain knowledge from the telemonitoring about management of exacerbations, which they may retain for variable periods of time. A washout period could, therefore, be 1 week, 1 month, 6 months or more according to individual variation, and during this period deterioration in overall clinical status could occur. For that carefully considered reason, a washout period was not included in the trial design.

An addition consideration is that the duration of monitoring and control periods was 6 months, which could be impacted upon by seasonal variations in exacerbations, despite randomisation of patients to each arm.

We used a second-generation telemonitoring system-this is a non-immediate or analytical decision system where the data transfer is synchronous with an automated algorithm. The team recognises important changes in the patient's condition but delays in response can occur if the system is only monitored at certain times. By comparison, third-generation systems have constant analytical and decision-making support in which monitoring centres are physician led, staffed by specialist nurses and have full authority to effect therapy changes 24 h per day, 7 days per week. We chose to use the Phillips Motiva system as it was available to us at the start of the trial and had been successfully deployed in the Whole System Demonstrator trial. While third-generation systems might offer advantages in theory, to date there is no evidence that one system is better than another in practice.

### Managing telemonitoring data

A key issue with monitoring systems is the generation of ‘too much information’ due to a lack of appropriately sensitive and specific algorithms. This continues to be one of the main challenges for telemonitoring in respiratory disease and is highlighted in our results by the number of out-of-range alerts and high number of ‘red’ alerts generated related to SpO_2_ levels. This was despite individualised setting of the target levels by the healthcare team. A similar finding of frequent sampling of physiological data prompting increased interventions was observed in the DOT-HF trial^23^ in which the use of an intrathoracic impedance monitoring device to closely track fluid balance in patients with heart failure resulted in an almost threefold increase in outpatient visits and 79% increase in heart failure admissions.

### Significance of telemonitoring and the future

Our results differ very significantly from those of Vitacca *et al*^5^ who showed in a group of patients with COPD and non-COPD patients that telemonitoring produced a reduction in healthcare activity. However, the Bilbao trial generated more telephone calls to patients in the telemonitoring group,^6^ while both the WSD trial^9^ and Edinburgh trial^10^ showed no reduction in healthcare activity. It is notable that the two largest trials to date of telemonitoring in heart failure^2^ ^3^ showed no change in healthcare utilisation. There is growing evidence that the impact of telemonitoring will depend on the integration of this process with existing care pathways, and links between the patient, GP, community and hospital teams. If there is a clearly identified pathway already operating and care has been optimised, telemonitoring may not have much to add, and as shown here may intensify workload by generating further healthcare visits to check telemonitoring data. However, in situations where the home network is less well established, telemonitoring may have more to contribute. Telemonitoring may be more relevant in patients who are widely dispersed geographically, for example. Although in TeleCRAFT we did not find that socially isolated patients benefitted more greatly from telemonitoring compared with patients with a home relative or carer. Furthermore, there may be advantages to using telemonitoring in patients who have poor access to hospital because of mobility problems, for example, housebound individuals or those with progressive conditions such as motor neurone disease/amyotropic lateral sclerosis (MND/ALS).

## Conclusions

The results of the TeleCRAFT trial suggest that the application of telemonitoring using a second-generation system in patients with chronic respiratory failure increased healthcare activity, without generating an improvement in quality of life for the patient. Consideration should be given to the investigation of targeting this resource to particular subgroups rather than an unfocussed widespread application in chronic respiratory disease.
